# Nutritional Quality Implications: Exploring the Impact of a Fatty Acid-Rich Diet on Central Nervous System Development

**DOI:** 10.3390/nu16071093

**Published:** 2024-04-08

**Authors:** Katarzyna Smolińska, Aleksandra Szopa, Jan Sobczyński, Anna Serefko, Piotr Dobrowolski

**Affiliations:** 1 Chronic Wounds Laboratory, Medical University of Lublin, Chodźki St. 7, 20-093 Lublin, Poland; katarzyna.smolinska@umlub.pl; 2 Department of Clinical Pharmacy and Pharmaceutical Care, Medical University of Lublin, Chodźki St. 1, 20-093 Lublin, Poland; aleksandra.szopa@umlub.pl (A.S.); jan.sobczynski@umlub.pl (J.S.); anna.serefko@umlub.pl (A.S.); 3 Department of Functional Anatomy and Cytobiology, Maria Curie Sklodowska University, Akademicka St. 19, 20-033 Lublin, Poland

**Keywords:** fatty acids and neurodevelopment, pediatric nutrition, central nervous system development, omega-3 fatty acids, neurodevelopmental disorders

## Abstract

Given the comprehensive examination of the role of fatty acid-rich diets in central nervous system development in children, this study bridges significant gaps in the understanding of dietary effects on neurodevelopment. It delves into the essential functions of fatty acids in neurodevelopment, including their contributions to neuronal membrane formation, neuroinflammatory modulation, neurogenesis, and synaptic plasticity. Despite the acknowledged importance of these nutrients, this review reveals a lack of comprehensive synthesis in current research, particularly regarding the broader spectrum of fatty acids and their optimal levels throughout childhood. By consolidating the existing knowledge and highlighting critical research gaps, such as the effects of fatty acid metabolism on neurodevelopmental disorders and the need for age-specific dietary guidelines, this study sets a foundation for future studies. This underscores the potential of nutritional strategies to significantly influence neurodevelopmental trajectories, advocating an enriched academic and clinical understanding that can inform dietary recommendations and interventions aimed at optimizing neurological health from infancy.

## 1. Introduction

The development of the nervous system in children is a sophisticated process that is influenced by a myriad of environmental factors, among which nutrition plays a critical role. Fatty acids (FAs), particularly long-chain polyunsaturated FAs (LC-PUFAs), have emerged as key dietary components because of their essential functions in neurodevelopment. These FAs are integral to the formation and maintenance of neuronal membranes, the modulation of neuroinflammation, and the facilitation of neurogenesis and synaptic plasticity. Despite the acknowledged importance of FAs in neurodevelopment, the precise mechanisms through which specific dietary FAs contribute to the development of the central nervous system (CNS) and their optimal levels during various stages of childhood remain an area of ongoing research.

The current literature delineates the significance of omega-3 FAs such as docosahexaenoic acid (DHA) and eicosapentaenoic acid (EPA) in supporting cognitive development and mental health [1,2,3]. However, there is a noticeable gap in the comprehensive review and synthesis of recent findings that articulate a broader spectrum of FAs, including omega-6 FAs and their ratios to omega-3 FAs, in the context of neurological development and function.

Our review article aims to consolidate the existing knowledge on the influence of a fatty acid-rich diet on CNS development in children, exploring both beneficial and potentially adverse effects. It critically assesses the current understanding from molecular biology to clinical outcomes, highlighting the essential roles of FAs in neurodevelopmental processes and their implications for dietary recommendations.

Moreover, this review identifies the need for further research in several key areas, including the impact of FA metabolism on neurodevelopmental disorders, the long-term effects of early-life nutrition on adult neurological health, and the development of age-specific dietary guidelines that optimize neurodevelopmental outcomes. By addressing these gaps, this review contributes to a more nuanced understanding of the relationship between nutrition and nervous system development and proposes suggestions for potential future research that could offer valuable input for the development of dietary guidelines and interventions.

In doing so, we refer to original studies and recent meta-analyses, such as those conducted by Lehner et al. (2021) and Verfuerden et al. (2020), which have begun to illuminate these complex interactions but also call for a deeper, more integrated approach to understanding the nutritional underpinnings of neurodevelopment [4,5]. Through a detailed examination of the current evidence and identification of research gaps, our review endeavors to advance the field of pediatric nutrition and neurodevelopment, setting a stage for future investigations to further elucidate the critical role of dietary FAs in the developing brain.

## 2. The Types of Fatty Acids

Fatty acids (FAs) are carboxylic acids comprising a long carbon chain and at least one carboxyl group; their links can be double bonds. Most naturally occurring FAs have an unbranched chain with an even number (4–28) of carbon atoms. FAs can be divided into four groups based on the number of carbon atoms in the aliphatic hydrocarbon chain. Short-chain, medium-chain, long-chain (LCFAs), and very-long-chain fatty acids (VLCFAs) contain up to 5 carbon atoms (C1–5), 6–12 carbon atoms (C6–12), 13–21 carbon atoms (C13–21), and more than 22 carbon atoms (C > 22), respectively. Another criterion used to categorize FAs is the presence of double bonds. FAs can be divided into a few groups: saturated FAs, monounsaturated FAs, and polyunsaturated FAs (PUFAs). Omega-3 and omega-6 FAs are subgroups of polyunsaturated fatty acids (PUFAs). They possess numerous double bonds, and the first contains three and six atoms away from the terminal methyl group in their aliphatic hydrocarbon chains for omega-3 and omega-6 FAs, respectively [6]. The most notable omega-3 FAs are all-cis-9,12,15-octadecatrienoic acid (α-linolenic acid—ALA), all-cis-5,8,11,14,17-eicosapentaenoic acid (EPA), all-cis-docosa-4,7,10,13,16,19-hexaenoic acid (DHA), and all-cis-6,9,12,15-octadecatetraenoic acid (stearidonic acid) [7].

## 3. Nutritional Importance of Fatty Acids

Essential fatty acids (EFAs), as their name suggests, are a group of fats that are crucial for maintaining human health. As EFAs cannot be synthesized in sufficient amounts by organisms, they must be supplied with food. The condensation of acetyl-CoA in cells facilitates the formation of palmitic acid, which contains 16 carbon atoms. In addition, through elongation and desaturation in the endoplasmic reticulum, the length of the aliphatic chains increases, and double bonds are introduced into the FA molecules. Although mammalian desaturases can insert double bonds at specific locations (Δ9, 6, 5, and 4), they cannot be added to positions beyond the tenth carbon of the fatty acid chain. As such, only ALA and linoleic acid (LA) are essential FAs for humans because they are precursors of other members of the omega-3 and omega-6 families, respectively [8].

In all major dietary sources, PUFAs are chemically bound to glycerol molecules in the form of triacylglycerols (TAGs) or diacylglycerols. Other chemical entities containing FAs include phospholipids (e.g., lecithin) and cholesterol esters [8]. Like other long-chain fatty acids (LCFAs), ALA is absorbed from the gut and enters circulation, where it is primarily esterified into triacylglycerols carried by chylomicron particles. These triacylglycerols are further hydrolyzed by lipoprotein lipase, which is expressed in the endothelium. Fatty acids from chylomicron remnants are processed in the liver and may reappear in the bloodstream as components of triacylglycerols (very-low-density lipoproteins) or phospholipids. Through these processes, dietary ALA can be made available to be incorporated into cell membranes and pools for storage, energy production, or conversion into long-chain omega-3 polyunsaturated fatty acids (PUFAs), which are believed to occur mainly in the liver [9]. It should be noted that, following absorption in the gastrointestinal tract, most ALA is catabolized via β-oxidation for energy production. Only a fraction of ALA is converted into two other important EFAs: EPA and DHA. In the human body, the rate of conversion of ALA to EPA and DHA has been reported to be 8–12% and <1%, respectively [10]. However, recent research has indicated that DHA synthesis rates cannot be as low as previously reported [11]. The conversion of omega-6 and omega-3 FAs is a competitive process because both utilize the same metabolic pathways. However, if the availability of LA exceeds that of ALA, metabolism of the former exceeds that of the latter. The conversion of ALA to EPA and DHA appears to be more efficient in women than in men [12]. Considering these biochemical properties, a well-balanced diet should not only contain a sufficiently high intake of omega-3 and omega-6 PUFA but also a proper proportion between them [13].

Genetic factors may also influence the ALA conversion rate, as many single nucleotide polymorphisms have been identified in the FA desaturase (Fads1 and Fads2) genes [14]. The significance of this genetic variability should be considered when preparing recommendations for dietary PUFA intake. For instance, the C allele of FADS1 rs174547, which plays a crucial role in the conversion of plant-based LC-PUFAs into VLC-PUFAs, is commonly found in Americans (59%), East Asians (57%), and Europeans (35%). However, it is rarely present in South Asian (14%) or African (2%) populations. Therefore, it has been proposed that a diet rich in ALA may benefit South Asian and African populations (who have limited access to seafood) because of the normal functioning of desaturase enzymes (absence of mutations) and efficient conversion of ALA. However, Americans may require direct supplementation with EPA and DHA because of the low conversion rate of ALA to longer-chain EFAs [15].

## 4. Dietary Sources of Fatty Acids

There are numerous recommendations concerning the optimal dietary intake of PUFA in a healthy diet [16]. According to the WHO, the minimum intake values for EFAs to prevent deficiency symptoms are estimated at the following levels: 2.5% of daily energy intake should be consumed as LA, and 0.5% of daily energy intake should be consumed as ALA. The recommended daily intake of EPA and DHA for adult males and non-pregnant and non-lactating adult females is 250 mg per day. Currently, there is insufficient evidence to establish a specific minimum intake of either EPA or DHA alone; therefore, both should be consumed. For pregnant and lactating adult females, the minimum intake for optimal adult health and fetal and infant development is 300 mg/day EPA + DHA with at least 200 mg/day DHA [17,18]. The National Institutes of Health recommends an adequate intake of 1.4 g of omega-3 FAs per day for male children aged >14 years and men. For women, pregnant women, and lactating women, the numbers should be 1.1 g, 1.4 g, and 1.3 g, respectively. Specific intake recommendations for EPA and DHA have not been established [19,20]. The European Food Safety Authority proposes that 4% of the total energy intake should be provided as LA, and 0.5% of the total energy intake should be provided as ALA, regardless of age, sex, and pregnancy status. While children up to the age of 1 year should consume 100 mg DHA daily, children older than 1 year and all adults should consume 250 mg DHA and EPA daily [21].

**Figure 1:**
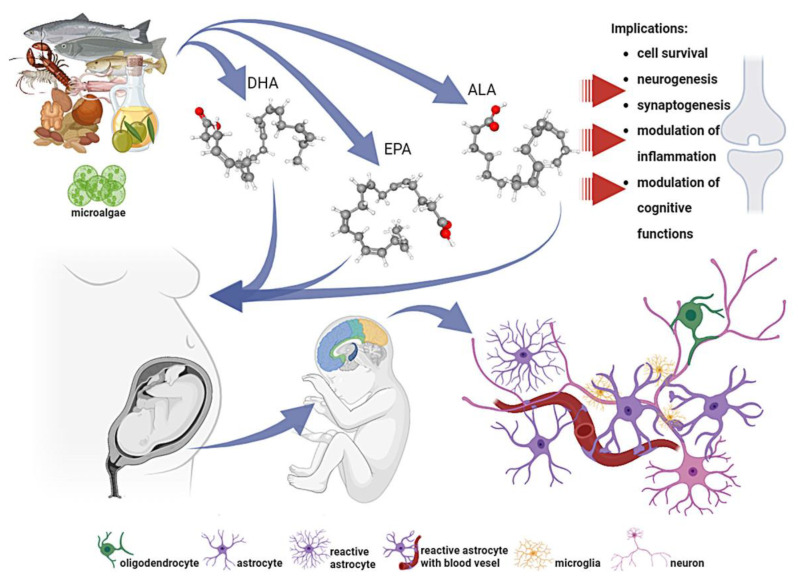
Food sources of omega-3 unsaturated fatty acids (DHA, EPA, and ALA) and their roles in central nervous system development. ALA, α-linolenic acid; DHA, docosahexaenoic acid; EPA, eicosapentaenoic acid. Created with BioRender.com (accessed on 1 March 2024).

Considering the metabolic pathway of PUFAs in cells, they can be provided by the consumption of fish rich in DHA and EPA or plant seeds or oils that contain ALA. PUFA-rich sources include certain seeds, nuts, and oils (Table 1) and, to a lesser extent, beans. The most common EPA and DHA sources are fish, especially species found in cold waters and seafood, meat, and eggs to a lesser extent (Figure 1). Other sources of PUFAs have been proposed. Mushrooms can contribute to the uptake of FAs with some advantages, such as their ubiquity, low caloric value, high fiber and protein content, and absence of cholesterol [22]. The biosynthesis of omega-3 PUFAs in algae has also been extensively studied, especially in commercial species [23]. Alternative herbal, microbial, and seaweed sources of DHA and EPA, which could be acceptable for vegans, have also been proposed [8,24].

Despite the fact that various scientific committees and advisory bodies have published their recommendations, the observed intake of omega-3 FAs in the population is lower than required. A recently published study on the American population showed that toddlers, children, and adolescents had significantly lower omega-3 FAs intake than adults and seniors. Moreover, females exhibited a lower intake of omega-3 FAs than males, and adult and senior women had a significantly lower intake than men in the same age groups. Women also consumed less fish than men. The estimated intake of omega-3 FAs in pregnant women did not differ from that in non-pregnant women, although pregnant women reported lower consumption of high-omega-3-FA-containing fish than non-pregnant women. Only 0.8% of the study population reported using EPA/DHA supplements, but this was associated with significantly higher EPA, DHA, and EPA + DHA intake than in non-supplement users. This indicates that some subgroups of the population may be at a higher risk for omega-3 FAs intake below the recommended levels [26].

## 5. Omega-3 Polyunsaturated Fatty Acids in Brain Health: Structural and Functional Impacts of Docosahexaenoic Acid on Neural Development and Homeostasis

In mammals, lipids account for approximately half of the dry weight of the brain, with 35% being omega-3 PUFAs. DHA constitutes over 40% of omega-3 PUFAs present in neuronal tissues, particularly in the gray matter. In contrast, EPA represents less than 1% of the total acid content of the brain [27]. FAs are critical constituents that determine the structural diversity of lipids and their function in the CNS [28]. In addition, FAs and their metabolites play crucial roles in maintaining brain homeostasis and affect several neural functions, including cell survival, neurogenesis, and synaptogenesis [29,30]. Most DHA accumulation in the brain occurs during the perinatal period, which is a period of significant brain development [31]. DHA, a significant component of neural membranes, makes for 30–40% of phospholipids in the gray matter of the cerebral cortex and photoreceptor cells of the retina. It plays a crucial role in regulating the physicochemical properties of synaptic membranes, such as fluidity, permeability, and viscosity, as well as in modulating the neurotransmission, gene expression, and activities of enzymes, receptors, and ion channels [32]. Brain development involves the incorporation of DHA into membrane phospholipids, particularly phosphatidylethanolamine, which leads to neurite outgrowth, synaptogenesis, and neurogenesis. In the hippocampus, omega-3 PUFAs exposure enhances synaptic plasticity by increasing synaptic protein expression, dendritic spine density, and long-term potentiation [33]. It augments glutamatergic synaptic activity accompanied by an increase in the expression of synapsin and glutamate receptor subunits [34]. DHA modulates the activity of glutamate transporters such as GLT1, GLAST, and excitatory amino acid transporter 3 (EAAC1) through extracellular Ca^2+^, Ca^2+^/calmodulin-dependent protein kinase II (CaM), and protein kinase C [33]. Cerebral endothelial and glial cells produce DHA and EPA from dietary sources and serve as DHA precursors. However, the capacity for endogenous synthesis or conversion of DHA is notably low [35]. Therefore, dietary DHA is the main source of DHA in the brain [36]. Although neurons cannot synthesize PUFAs, they can incorporate them into their membranes [37] (as shown in Figure 1).

In addition to the well-documented roles of DHA in enhancing cognitive and visual function, recent studies have elucidated its synergistic interaction with phosphatidylserine (PS) in membrane dynamics, which is crucial for brain health. Sinclair (2019) highlighted the integral role of PS, predominantly localized on the cytoplasmic side of neuronal cell membranes, in facilitating the action of signaling proteins that underpin neuronal survival, neurite growth, and synaptogenesis. DHA not only promotes the synthesis of PS but also leads to an expansion of the PS pool in neuronal membranes, thus influencing PS-dependent signaling pathways vital for optimal neuronal function. This interaction underscores the multifaceted role of DHA in brain health, extending beyond its direct action to include significant effects on membrane lipid composition and function, thereby enhancing signal transduction processes essential for cognitive and neurological health [38].

Glial cells are typically divided into three primary types: astrocytes, microglia, and oligodendrocytes. Each of these cell categories plays a crucial role in maintaining neuronal health in their vicinity [39]. Astrocytes are the primary source of DHA, which is synthesized (essential) within the central nervous system. Although most DHA in the CNS is obtained from dietary sources (non-essential), the synthesis of DHA from ALA by astrocytes in response to various stimuli is crucial for neuroinflammation and cell survival [40]. DHA diminishes microglial-induced inflammation by inhibiting the nuclear factor-κB (NF-κB) and mitogen-activated protein kinase (MAPK) pathways [41], whereas DHA deficiency increases the expression of pro-inflammatory cytokines such as interleukin-6 (IL-6) and tumor necrosis factor α (TNF-α) [42]. Research has demonstrated that fatty acid metabolism plays a crucial role in regulating astrocyte function under both normal and abnormal conditions. Some studies have shown that the synthesis of fatty acids by astrocytes is necessary for neuronal differentiation during development. In particular, it has been reported that an increase in albumin during this phase triggers the expression of sterol regulatory element-binding protein 1 (SREBP-1), leading to the accumulation of oleic acid, a monounsaturated fatty acid (MUFA). Astrocytic oleic acid is transferred horizontally to phosphatidylcholine (PC) and phosphatidylethanolamine (PE) in neurons, thereby promoting neuronal differentiation. Oligodendrocytes synthesize sphingomyelin, which stimulates myelination. These data suggest that fatty acid metabolism is a key factor in astrocyte function under physiological and pathological conditions [38,43].

DHA is a crucial precursor of various mediators, including protectins and resolvins, which are signaling molecules that play a role in inflammation [44]. It can also be metabolized to docosahexaenoylethanolamide (DEA), the main endogenous ligand of orphan adhesion G-protein-coupled receptor 110 (GPR110, ADGRF1) in the brain [34,45]. Activation of the GPR110 receptor by the endogenous ligand synaptamide promotes neurogenesis, neurite growth, and synaptogenesis in the developing brain through cAMP signal transduction [46]. GPR40/FFAR1 is a free FA (FFA) receptor expressed in different regions of the CNS, including the cortex, hypothalamus, and spinal cord [47]. DHA is a GPR40/FFAR1 agonist. Experimental research has demonstrated that GPR40/FFAR1 plays a crucial role in mediating the effects of DHA on a variety of physiological activities, such as insulin secretion, regulation of hormone secretion in the digestive system, taste perception, and bone remodeling [47,48]. Activation of the GPR40 receptor in the CNS has been linked to pain control and the regulation of cognition and emotional behavior [49,50]. Although the positive impact of DHA on the central nervous system, particularly at the molecular level, is well established, the specific molecular pathways involved in its neurotrophic and neuroprotective effects are not fully understood. Identifying these pathways may pave the way for innovative therapeutic approaches for the treatment of CNS diseases [51]. In our comprehensive review of fatty acids crucial for neurodevelopment, *n*-3 docosapentaenoic acid (DPA) is also a noteworthy inclusion. Despite its relative obscurity compared to its *n*-3 PUFA counterparts EPA and DHA, *n*-3 DPA has a significant, albeit understated, role in the brain and other tissues. Ghasemi Fard et al. (2021) illuminated the nuanced biological functions of *n*-3 DPA, underscoring its potent effects which may not be immediately apparent—akin to the largely submerged part of an iceberg. This minor omega-3 fatty acid, which is less abundant than DHA and EPA, is intricately involved in the biosynthesis of specialized pro-resolving lipid mediators (SPMs) such as resolvins, maresins, and protectins. *n*-3 DPA mediators are critical for reducing inflammation and regulating immune reactions, which underscores their significant and extensive impact on neurodevelopment and overall health [52]. The importance of *n*-3 DPA should be highlighted to explore its role further.

## 6. Impact of Malnutrition on Neurodevelopment and Child Health

Malnutrition encompasses deficiencies and excess nutrient intake, an imbalance of essential nutrients, and impaired nutrient utilization, as defined by the World Health Organization (WHO). UNICEF’s 2022 report highlights that approximately 2.4 billion individuals, predominantly women and rural residents, lack access to adequate food supplies, with more than 200 million children suffering from various forms of malnutrition. This issue is particularly critical for children under the age of five, including newborns, where malnutrition significantly contributes to long-term neurological disabilities and impaired developmental outcomes. Increasing attention has been paid to the role of specific nutrients such as DHA in supporting the complex process of neurodevelopment in early childhood. Particularly, in preterm infants who are at a heightened risk of developmental challenges, early nutritional interventions have shown promising results. A recent study demonstrated that neonatal supplementation with DHA in preterm infants is linked to improved intelligence quotients at 5 years, underscoring the long-term cognitive benefits of targeted nutritional support during critical developmental windows [53]. This finding aligns with further discussion of the crucial impact of malnutrition and specific nutrient deficiencies during the prenatal and neonatal periods on subsequent child health and cognitive development. Despite widespread concerns about the adequacy of dietary intake of omega-3 fatty acids, particularly DHA, and its implications for brain health, recent research has provided a nuanced perspective on this issue. Sinclair et al. (2022) meticulously explored the evidence for dietary-induced DHA deficiency in human brains, challenging prevailing assumptions about the prevalence of such deficiencies. Their review also elucidated the critical periods for DHA accrual in the brain, notably during fetal and postnatal development, and identified specific populations that might be at risk owing to dietary patterns. This comprehensive analysis not only highlights the complex interplay between diet and brain DHA levels but also highlights the significant gaps in our understanding, particularly concerning the direct evidence of DHA deficiency in human brain tissue. This research underscores the importance of continued investigation into dietary needs for optimal brain function and the potential consequences of deficiency [54]. Moreover, as suggested by Sinclair et al. (2022), the prevalence of omega-3 PUFA deficiency in the human brain is not well documented owing to insufficient data, particularly from populations that are likely to be deficient. As shown in their review, (1) most of the existing research focuses on Western countries, where dietary omega-3 PUFA deficiency is less common; (2) vegetarians, especially vegans, tend to have lower levels of DHA in their plasma, blood, and tissues because their diets are typically rich in LA and poor in ALA and LC-PUFAs (significant signs of omega-3 PUFA deficiency were generally not found in these groups despite their dietary restrictions); and (3) there is evidence of potential omega-3 PUFA deficiency in a few specific cases involving newborns: Hindu vegetarian mothers in London had infants with high levels of omega-3 PUFA deficiency markers in cord arterial tissue, infants in the U.K. and Australia showed signs of deficiency when fed formulas low in omega-3 PUFAs, and mothers from northern Sudan had very low DHA levels in their milk. As the authors concluded, the lack of global data on the status of DHA and others PUFAs in brain tissue from risk regions suggests an urgent need for research in populations at risk of its deficiency [54]. Notably, low birth weight associated with malnutrition has been linked to long-term neurological disabilities and delayed language development [55]. Additionally, congenital defects such as neural tube abnormalities may arise from selective deficiencies in essential micronutrients during prenatal development [56]. The “Barker hypothesis” suggests that low birth weight is a crucial risk factor for chronic diseases, including systemic arterial hypertension and chronic renal insufficiency [57]. Nutrient deficiencies during prenatal development, particularly tryptophan, folate, and B vitamins, may result in irreversible alterations in the brain [58]. B vitamins, which are essential for healthy brain development, highlight the critical role of nutrition in cognitive function and overall health [59]. Fatty acids, which are pivotal for normal fetal growth, underline maternal nutritional status as a determinant of health outcomes [60]. Evidence from animal studies indicates that omega-3 fatty acid deficiency during pregnancy leads to visual and behavioral deficits that are irremediable postnatally [61]. Thus, addressing undernutrition, including the inadequate intake of proteins, vitamins, and essential fatty acids, especially in pregnant women in developing countries, has emerged as a paramount public health concern [62].

## 7. Cognitive Dysfunction in Children and Fatty Acids

Cognitive impairment encompasses various facets of intellectual and cognitive performance, such as working memory, attention, and executive function [63]. Cognitive function is fundamentally linked to both instinctive and learned behaviors and forms the foundation for successful education within and beyond the classroom during childhood and teenage years. If cognitive skills are compromised, it may hinder a child’s ability to function autonomously in adulthood or lead to behavioral issues that obstruct learning and its practical application [64]. It is estimated that this problem, which can influence learning and intellectual abilities, is present in approximately 2–5% of the pediatric population. Cognitive disorders in children can be associated with a range of diseases and conditions including but not limited to (1) neurodevelopmental disorders, including autism spectrum disorder, attention-deficit/hyperactivity disorder (ADHD), learning disorders, intellectual disability, and communication disorders that affect brain development and functioning; (2) genetic conditions (Down syndrome, Fragile X syndrome, and Williams syndrome); (3) neurological disorders (epilepsy, cerebral palsy, traumatic brain injury, and brain tumors); (4) infectious diseases (meningitis and encephalitis); (5) metabolic disorders (thyroid dysfunction and diabetes); (6) environmental exposures (lead poisoning and prenatal exposure to alcohol or drugs); (7) psychiatric conditions (depression and anxiety disorders); (8) chronic illnesses (cancer and treatments like chemotherapy, sickle cell disease, and chronic kidney disease); and (9) nutritional deficiencies (iron-deficiency anemia, malnutrition, and essential FA deficiencies) [64,65,66,67,68,69,70,71].

More than 20 years ago, a positive correlation between omega-3 PUFAs during pregnancy and cognitive functions in childhood, such as sequential processing and intelligence quotient (IQ), was observed [71,72,73]. Furthermore, recent studies have indicated a link between the levels of PUFAs in mothers during pregnancy and their children’s cognitive abilities, including intelligence [74]. Nevertheless, in the field of pediatric nutrition and cognitive development, research findings regarding the impact of omega-3 LC-PUFA supplementation are mixed. However, most publications indicate the benefits of DHA supplementation in populations at risk for FA deficiency. For example, Stonehouse [75] pointed out that people who typically consume insufficient amounts of omega-3 LC-PUFAs, undernourished children with limited literacy skills, and older people experiencing the early stages of memory and thinking disorders may benefit the most from increased DHA intake. Moreover, neonates delivered significantly preterm, particularly at or before the juncture marking the commencement of the final trimester of gestation, exhibited diminished DHA concentrations within neural tissues [31,76]. Two meta-analyses conducted in 2018 showed that this population was at an elevated risk of cognitive function deficits [77,78]. Furthermore, assessments of intellectual capacity, as determined by IQ evaluations, typically reveal a decrement of 10–12 points in comparison with infants born at full term [77,79].

Several observational studies have consistently shown a relationship between the fish consumption habits of pregnant women and the neurological and cognitive development of their offspring, according to various sources [80,81,82,83]. However, comprehensive analyses, including systematic reviews and meta-analyses of clinical trials that supplemented omega-3 PUFAs during pregnancy, breastfeeding, and in infant formulas, have yielded inconsistent results [84,85,86,87,88,89]. In a comprehensive systematic review and meta-analysis of randomized control trials, Lehner et al. [4] investigated the impact of LC-PUFA supplementation, primarily consisting of DHA and EPA, on cognitive outcomes in children of pregnant and lactating mothers. By examining 22 publications corresponding to 11 trials, they found no significant relationship between DHA/EPA supplementation and any of the assessed cognitive parameters. These findings are consistent with the outcomes of previous reviews and meta-analyses on this subject [89,90,91]. In addition, the results of a meta-analysis performed by Emery et al. [92] showed that supplementation with either DHA- or EPA-rich omega-3 PUFA did not lead to improvements in attentional capacities in typically developing children and adolescents, indicating that omega-3 PUFA do not enhance performance in tasks requiring vigilant attention, a critical cognitive function that allows maintenance of attention, particularly in redundant or intellectually unchallenging situations [92]. Similarly, Nevins et al. [93] conducted a systematic review to examine the relationship between omega-3 FA supplementation during pregnancy and/or lactation and neurodevelopment in children. They enrolled 33 articles from 15 randomized controlled trials and 1 prospective cohort study. Among the randomized controlled trials that administered dietary omega-3 FA supplements (ranging from 200 to 2200 mg/day of DHA and 0 to 1100 mg/day of EPA for approximately 20 weeks) during pregnancy, only five reported at least one positive outcome, indicating that supplementation led to a 6–11% improvement in cognitive development measures in infants or children. Most of the eight studies uncovered at least one outcome that failed to achieve statistical significance. These studies typically fell short of adequately representing populations with lower socioeconomic status and adolescents, as they displayed a scarcity of racial and ethnic diversity among the study participants. Based on the findings of this review, the authors concluded that there is a limited body of evidence to suggest that supplementation with omega-3 fatty acids during pregnancy might positively affect a child’s cognitive development [93].

The literature also included meta-analyses and systematic reviews aimed at assessing the impact of PUFAs on cognitive function in older children and adolescents. A systematic review conducted by Verfuerden et al. [5] investigated the cognitive outcomes in children aged 2.5 years or more who were randomized as infants to receive formula with added LC-PUFAs compared to those who received formula without these supplements. Nonetheless, the overall quality of evidence from this meta-analysis was considered low because of the significant variability in the study results, poor follow-up rates, and signs of selective reporting in the publications. The long-term effects of LC-PUFA supplementation on the cognitive development of both term and preterm infants remain highly uncertain, with possibilities ranging from considerable benefits to significant harm. In light of these findings, the authors concluded that supplementation of infant formula with LC-PUFAs cannot be recommended until more reliable evidence is available to rule out the potential for long-term adverse effects [5]. A review by van der Wurff et al. [94] aimed to investigate whether a certain omega-3 index level and minimum daily omega-3 LC-PUFA dose are required to improve cognition in 4- to 25-year-olds. An examination of 33 studies revealed more frequent observation of cognitive improvements when there was an increase in the omega-3 index beyond 6%. In the case of children with typical development, half of the studies that provided a daily dose of 450 mg of DHA and EPA reported enhanced cognitive function. However, no specific threshold has been identified for children with disorders. On this basis, they concluded that a daily intake of 450 mg DHA and EPA, along with an elevation in the omega-3 index above 6%, appeared to enhance cognitive effects in children and teenagers [94]. In addition, Kadosh et al. [95] highlighted the significance of nutrient roles in the cognitive development of infants and young children, thereby emphasizing the growing importance of polar lipids. Their review elucidated how the distinct contributions of nutrients such as PUFAs and their synergistic interactions with other micronutrients and macronutrients as well as their organization within the food matrix are vital for proper neurocognitive development [95]. A meta-analysis by Emery et al. [92] aimed to expand our understanding of the effects of omega-3 supplementation on cognitive test scores in young individuals. This study included 29 published randomized controlled trials that examined the influence of omega-3 PUFAs on individuals from birth to the age of 25 years, a threshold commonly considered as the age of complete brain development. Quantitative analysis did not find evidence of a significant effect of omega-3 PUFA supplementation on youth performance in specific cognitive domains. Nonetheless, certain cognitive domains, notably long-term memory (specifically recall), working memory, and problem-solving abilities, showed potential improvements with supplementation of EPA-dominant omega-3 PUFA formulations. In contrast, DHA-rich omega-3 PUFA formulations did not exhibit any beneficial effects on cognitive domains, a finding that persisted even when analyses were restricted to the most favorable study results [92].

A recent analysis of data from a substantial cohort study (17,641 children and teenagers) including 8656 girls and 8985 boys, prepared by Lehner et al. [96], aimed to explore the link between fish consumption and cognitive school performance in school-aged children and adolescents. Their research revealed a significant correlation, demonstrating that consuming 8 g of fish daily increased the likelihood of improving final grades in German (odds ratio 1.193; 95% confidence interval 1.049–1.358) and mathematics (odds ratio 1.16; 95% confidence interval 1.022–1.317) compared to negligible or no fish consumption. This finding aligns with prior studies suggesting a beneficial effect of fish intake on academic performance [97]. The study found that the relationship between fish consumption and cognitive school performance was not linear but instead showed a reduction in the positive effect at higher levels of fish consumption. This resulted in the identification of a U-shaped curve that demonstrated the association between the two variables among the children in the cohort [96]. The Seychelles Child Development Study Nutrition Cohort 2, which involved 1237 children aged 7 years and utilized a comprehensive neurodevelopmental test battery, evaluated various domains such as cognition, executive function, and language skills. Although some indicators of improved neurodevelopmental performance were observed with higher maternal omega-6/omega-3 PUFA ratios, these results were not statistically significant after accounting for multiple comparisons [98].

There are also reports on the impact of various types of diets, including vegetarian, on the level of PUFAs in children’s bodies and cognitive function. Studies have indicated that women following a vegetarian diet exhibit DHA levels that are reduced by 20–40% compared to women who consume a diet that includes meat [99]. Additionally, studies have demonstrated that vegan mothers tend to have breast milk with lower DHA content than mothers who include animal products in their diet [100]. Crozier and colleagues [101] carried out a prospective observational study to explore if vegetarianism during pregnancy affects the maternal nutritional status, particularly fatty acids, and cognitive function in children aged 6–7 years. This study included 3158 pregnant women aged 20–34 years and their children. The researchers assessed the children’s cognitive function, including visual working memory, rule learning and cognitive flexibility, working memory, and impulsivity and decision making, at the age of 6–7 years. The findings of this study suggest that a vegetarian diet during pregnancy is not harmful to a child’s neurocognitive development as long as the mother’s nutrient levels critical for neurological growth are maintained within normal limits [101].

The discrepancies observed in the aforementioned studies are often ascribed to differences in research design, such as the number of participants, amount of supplement given, length of time the supplements were taken, and methods used to evaluate outcomes. Relatively few investigations have been conducted into the long-term impact on children’s development. Table 2 presents the main results.

A correlation between omega-3 PUFA intake during pregnancy and improved cognitive function in children has been highlighted, although research on supplementation with long-chain omega-3 PUFAs, particularly DHA, has yielded mixed results. Although some studies have found potential benefits, especially for populations at risk for nutritional deficiencies, others have shown no significant improvement in cognitive outcomes. This inconsistency is also seen in research focusing on older children and adolescents, with some studies suggesting that higher omega-3 intake could be beneficial, whereas others found no substantial impact. Research has also shown that fish consumption by pregnant women is positively associated with children’s cognitive and school performance; however, this relationship is not linear. Research has also addressed the issue of dietary choices such as vegetarianism, indicating that while vegetarian diets may lead to lower DHA levels, they are not necessarily harmful to neurocognitive development if essential nutrients are preserved. Discrepancies in the study outcomes were attributed to differences in the study design and methodology. The overarching conclusion from previous studies indicates that although there is some evidence supporting the cognitive benefits of omega-3 FAs, especially in populations with nutritional deficits or specific genetic backgrounds, the overall evidence remains inconclusive. There is a need for more rigorous and diverse research to establish clear guidelines for omega-3 supplementation and its long-term impact on cognitive development in children and adolescents. The potential benefits must be weighed against the lack of consistent evidence and possibility of long-term adverse effects.

## 8. Polyunsaturated Fatty Acids in Neurodevelopmental Disorders: Therapeutic Potential and Clinical Insights

PUFAs play a critical role in several neurodevelopmental conditions including ADHD, autism, major depressive disorders, and anxiety. They are not the only factors that trigger the development of the above-mentioned diseases; they may also contribute to other (genetic, environmental, and anatomical/neural) factors and may have a modulatory role in the onset and severity of a given disorder [117]. Certainly, there is some therapeutic potential for PUFAs in neurodevelopmental conditions. Some studies have supported the role of PUFAs in alleviating hyperactivity, irritability, impulsivity, and aggressive behavior, which are the adverse symptoms of ADHD and/or autism. An improvement was observed in relation to children’s attention, academic performance, and parent- and teacher-rated behavior [118]. However, most meta-analyses that have assessed the beneficial effects of omega-3 FAs in patients with neurodevelopmental conditions have yielded ambiguous conclusions. First, the data were still sparse. Furthermore, differences in the methodology used in individual clinical trials, such as variable doses of DHA and EPA, different proportions of their combinations, wide range of intervention periods (i.e., from several weeks to several months), sample size, inclusion and exclusion criteria, concomitant medications, and heterogeneity in measuring the potential effect, may be responsible for the lack of significance of the analyzed outcomes. The literature suggests that patients with dietary deficiencies may benefit from PUFAs [119]. Supplementation with PUFAs is usually well tolerated and not associated with serious adverse reactions.

### 8.1. ADHD

Behavioral symptoms of ADHD, the onset of which is usually observed in children below seven years of age, include inattention, hyperactivity/impulsivity, aggression, learning problems (in reading, writing, or mathematics), executive functioning, and poor relations with other people. In a systematic review and meta-analysis based on seven studies, Chang et al. (2018) demonstrated that young people with ADHD have lower levels of total omega-3 FAs, EPA, and DHA in red blood cells [120]. A previous meta-analysis carried out by Hawkey and Nigg (2014) also reported significantly reduced overall blood levels of omega-3 FAs in patients with ADHD compared with the healthy population [121]. Lower levels of omega-3 FAs have also been detected in the cellular membranes of both adults and children with ADHD [122]. These abnormalities seem to correlate with ADHD symptomatology [123]. Furthermore, an association between impulsivity and omega-3 FA deficiency (due to for example their insufficient intake, rapid metabolism, or disturbances in the conversion of short-chain to long-chain acids) and hyperactivity has been observed [124]. Thus, many authors have assumed that increasing the intake of omega-3 FAs in individuals with ADHD (in a regular diet or by supplementation) would improve the symptoms of this disease. The relationship between omega-3 FA status and the development of ADHD may be due to its influence on cellular phospholipid membranes, dopamine cortical neurotransmission, and inflammatory processes [125].

The standard treatment for patients with ADHD involves pharmacotherapy with amphetamine-type stimulants (i.e., methylphenidate, atomoxetine, dexamphetamine, and lisdexamphetamine) and behavioral therapy. It is important to recognize that the National Institute for Health and Care Excellence (NICE) guidelines for ADHD diagnosis and management do not advise or suggest dietary FA supplementation for treating ADHD in children and young people [126]. Based on the guidelines provided by the World Federation of Societies of Biological Psychiatry (WFSBP) and the Canadian Network for Mood and Anxiety Treatments (CANMAT), it is not currently recommended to use omega-3 fatty acids (ranging from 120 to 1200 mg) as a monotherapy or as an adjunctive treatment for children with ADHD, as per the clinical guidelines [127]. This recommendation was developed based on the outcomes from one statistically significant meta-analysis (taking into account seven trials/comparisons and 534 participants) and several negative/null randomized clinical trials, with 344 participants. Preparations with a higher amount of EPA than DHA alone could be more effective (although the results did not demonstrate a clear dose-response association), and children with fatty acid deficiency may benefit more from such supplementation. According to the same guidelines, omega-9 FAs (at doses of 2–3 g), which are found in evening primrose oil, are not recommended for monotherapy or adjunctive use in children with ADHD.

However, several authors have claimed that supplementing children with ADHD with long-chain marine omega-3 FAs may be beneficial for the improvement of ADHD symptoms, and such supplementation does not seem to be associated with any harm to health. The available literature suggests that the intake of both omega-3 and omega-6 FAs may have beneficial effects on the overall symptoms of ADHD as well as on cognitive performance, inattention, and hyperactivity, although the impact is usually either small or statistically insignificant [120,128,129]. The results of 13 out of 18 clinical trials considered in the critical review by Rosi et al. [130] demonstrated that PUFAs (DHA, EPA, ALA, and/or others) administered to children and adolescents with ADHD as monotherapy or standard pharmacotherapy contributed to the improvement of their sympathology, which was measured with diverse scales (i.e., Conners’ Parent Rating Scale, Parent-rated ADHD Rating Scale, Clinical Global Impression-Severity-Attention-Deficit/Hyperactivity Disorder Scale, Child Behavior Checklist, Continuous Performance Test, Strengths and Difficulties Questionnaire, Child Health Questionnaire, and others). However, it was not observed that any positive changes were linked to a specific behavioral or cognitive characteristic. In accordance with the findings of a recent systematic review and meta-analysis of clinical trials conducted by Händel et al. [131], there is no evidence to suggest that PUFA supplementation has a positive impact on the primary symptoms of ADHD, behavioral issues, or quality of life in children and adolescents. These conclusions were based on the results of 31 randomized controlled clinical trials involving 1755 participants [131]. In a systematic review of randomized clinical trials, Abdullah et al. [132] indicated that there is little evidence to suggest that omega-3 supplementation provides any benefit to ADHD symptoms in children and adolescents. This conclusion was based on the results of six studies from 2009–2017 [132]. In fact, in most of them, omega-3 supplementation had beneficial effects on ADHD symptoms measured by Conners’ rating scales, but they were not statistically significant. Two studies revealed sex-dependent results, that is, a greater improvement in males than in females. According to a narrative review by D’Helft et al. [133], the administration of omega-3 FAs as monotherapy may not be sufficient to treat patients with ADHD. However, the addition of gamma-linolenic acid (GLA) (i.e., omega-6 FAs with anti-inflammatory potential) to EPA and DHA may augment the effects of these omega-3 FAs [133]. Data from the literature suggest that EPA:DHA:GLA in a 9:3:1 ratio may improve ADHD symptoms. The authors explained that, most probably in the presence of GLA, omega-3 acids prevented the accumulation of arachidonic acid in the serum. Previous systematic reviews by Bloch and Qawasmi [129] and Chang et al. [120] demonstrated better omega-3 FAs activity when administered at higher dosages (e.g., EPA at a dose of ≥500 mg produced a significant effect) [120,129]. In addition, the use of PUFAs as an add-on treatment to methylphenidate may potentiate the effects exerted by the latter, and such a combination has an acceptable safety profile [134,135,136]. The results of the most important studies that were carried out during the last five years, evaluating the effects of omega-3 FA in children and adolescents with ADHD, are presented in Table 3. The outcomes of the randomized placebo-controlled trial by Dopfner et al. (2021) (not mentioned in Table 3) also support the positive effects of omega-3/omega-6 FA supplementation on ADHD symptoms in preschool children [137].

It should be emphasized that in the clinical trials listed in Table 3 as well as in clinical trials analyzed in the above-mentioned meta-analyses and systematic reviews, participants were given various PUFAs, including EPA and DHA acids (most frequently), but also LA, omega-6 acids (i.e., GLA, LA), or precursors of both omega-3 and omega-6 FA-conjugated LA. Omega FAs were the only treatment, or they were concomitantly used with pharmacological or vitamin (A, D, or E) supplementation. Similarly, several distinct methods of evaluating results were used, such as various questionnaires/ratings, cognitive tasks, and functional magnetic resonance imaging (fMRI) assessment.

According to a panel of experts on ADHD (see [119]), if the decision to give omega-3 FAs to patients with ADHD arises, dietary supplements should contain both EPA and DHA (at least 750 mg/day) as well as vitamin E, which prevents the oxidation of FAs. Such supplementation should be administered for at least 12 weeks. However, each patient should be assessed first in relation to ADHD severity, treatment history, and benefit–risk balance of FA intake. Moreover, PUFA intake cannot be considered an alternative to evidence-based medications. According to the preliminary clinical guidelines for children with ADHD developed by Chang and Su [154], the recommended dosage of DHA and EPA is 750 mg/day. However, patients with a high level of inflammation or low levels of omega-3 acids should be administered 1200 mg/day. The recommended duration of supplementation was 16–24 weeks (for inattention) or 52 weeks (for behavioral changes). For safety reasons, the authors recommend routine blood tests for fasting glucose, cholesterol, HDL, LDL, and TG every 6–12 months [154].

Data regarding the effects of omega-3 FA supplementation in pregnant women on the development of ADHD in children are ambiguous. According to Kohlboeck et al. [155], higher concentrations of DHA in cord blood serum were associated with a decreased level of hyperactivity and inattention in children when they were 10 years old. Some studies have reported promising results for eating fatty fish (as a source of EPA and DHA) [156,157] or DHA supplementation [158,159] during pregnancy to prevent the development of hyperactivity in children, whereas others have not demonstrated any significant effects of such treatments [160]. Surprisingly, Gould et al. [161] reported that DHA supplementation might have a negative effect on the behavioral functioning of children whose mothers regularly took omega-3 FA before delivery. A total of 543 parent–child pairs were included in a follow-up study of a multicenter, double-blind, randomized controlled trial conducted on women with a singleton pregnancy who took 800 mg of DHA per day for several weeks, starting from trial entry (less than 21 weeks of gestation) until birth [161]. The study revealed that the scores on various assessment scales, including the BRIEF Global Executive, Behavioral Regulation, and Metacognition Indexes; the Shift, Inhibit, Monitor, Working Memory, and Organization of Materials scales; the Conners 3 ADHD index; the SDQ Total Difficulties score; the Hyperactivity/Inattention score; and the Peer Relationship Problems score were lower in children whose mothers were assigned to the intervention group than in those whose mothers were assigned to the control group.

### 8.2. Autistic Spectrum Disorders

The disease, which occurs during the first years of life, is usually characterized by an impairment in social-communicative skills (i.e., difficulties with verbal and non-verbal communication, problems with recognizing and expressing emotions, being overwhelmed in social situations, etc.) and restricted, repetitive behaviors (e.g., repetitive body movements or motions with objects, specific interests, ritualistic behaviors, resistance to change, etc.). Individuals with autism sometimes have impaired language skills or intellectual disabilities. Therefore, when assessing the efficacy of a given treatment in autistic spectrum, different parameters may be taken into consideration, including autism symptom severity, maladaptive behaviors, social interaction, communication, psychopathology, behavior, and sleep in toddlers. The association between low levels of PUFAs (DHA, EPA, and total omega-3) in the plasma and an elevated omega-6:omega-3 ratio, the development of autistic spectrum disorder, and the severity of its symptoms has been known for years [125,162,163]. Furthermore, Lyall et al. [164] and Huang et al. [165] suggested that an elevated intake of PUFAs during pregnancy may diminish the risk of developing autism in children, whereas low intake of PUFAs during the last months of pregnancy may be a risk factor [164,165]. Martins et al. [125] in their recent review also highlighted the problem of a low dietary intake of omega-3 acids (as DHA supplementation or with fish) in pregnant women in the context of the neurodevelopment of their children (cognitive function, language and communication skills, social behavior, and others). According to Graaf et al. [166], a higher maternal omega-6/omega-3 FA ratio may be associated with the development of autistic traits in offspring. Several drugs are used for the pharmacotherapy of autism, including risperidone, aripiprazole, methylphenidate, atomoxetine, selective serotonin reuptake inhibitors, amitriptyline, and loxapine. In fact, they do not cure the disease but can alleviate some symptoms [163]. Therefore, new treatment strategies for autism have been investigated, including various dietary interventions [167]. One novel approach is supplementation with omega-3 FAs. Tauri et al. [118] in their narrative review focused on randomized clinical trials published between 2007–2021 that evaluated the potential beneficial effects of polyunsaturated fatty acids in children with autism spectrum disorder. Outcomes from five out of ten studies confirmed the positive effects of such a supplementation (particularly in relation to social communication, hyperactivity, irritability, and stereotypy), whereas others did not demonstrate any significant activity when measured by several different scales (such as the Aberrant Behavior Checklist, Pervasive Developmental Disorders Screening Test II, Brief Infant Toddler Social and Emotional Assessment, Social Responsiveness Scale, Pervasive Developmental Disorder Behavioral Inventory, and others). Based on their literature review, the authors suggested that the children of obese women could be a subpopulation of patients with autism who benefit the most from polyunsaturated fatty acid supplementation [118]. Therefore, additional intake of omega-3 FAs could be particularly recommended for children with autistic spectrum disorders and abnormal FA metabolism as an etiological factor of the disease. A previous meta-analysis by Fragaus et al. [168] demonstrated the positive effects of omega-3 FAs as a dietary intervention in relation to general autistic psychopathology, restricted repetitive behaviors, stereotypies, hyperactivity, and language function. Based on the results of 15 case–control studies (with 1193 participants) and four randomized control trials, Mazahery et al. [169] also assumed that the intake of omega-3 FAs has the potential to partially improve some symptoms of autism. In a recent meta-analysis, de Andrade Wobido et al. [170] demonstrated that omega-3 acids can significantly improve both social interaction and repetitive and restricted interests when compared with placebo-treated patients. It has been suggested that the impact of omega-3 and omega-6 FAs on neurite outgrowth (via modification of the neuronal cytoskeleton, expansion of neuronal membranes, or influence on vesicular fusion), synaptic transmission, or release of neurotransmitters may be responsible for behavioral and cognitive improvement in patients with autism [171]. In contrast, a systematic review with meta-analyses carried out by De Crescenzo et al. [172] did not provide evidence that supplementation with EPA or DHA may improve ASD symptoms of autistic spectrum disorder in children and adolescents. The authors included nine studies with 405 participants [172]. Similar conclusions were drawn in two systematic reviews by James et al. [173] and Horvath et al. [174]. Bozzatello et al. [175], Veselinovic et al. [176], and Pancheva et al. [177] drew attention to the fact that clinical studies that have evaluated the effects of omega-3 FAs on the symptoms of autism are highly contrasting. According to a review by Bozzatello et al. [175], four trials out of eight supported their beneficial role, whereas four did not indicate any significant clinical improvement. In positive studies, dietary intervention had beneficial effects on the following parameters: stereotypy, hyperactivity (impulsivity, distractibility, and disobedience), and social withdrawal. Furthermore, baseline levels of FAs in the blood are predictive factors for clinical response to omega-3 supplementation [175]. According to a review by Pancheva et al. [177], 7 out of 12 trials revealed that omega-3, omega-6, and/or omega-9 FAs may have therapeutic potential in autistic spectrum disorder when administered for several weeks. Positive changes in pediatric patients were noted in relation to sleep, behavioral and verbal activity, social communication, and motivation [177]. Jiang and colleagues [178], who reviewed 10 studies evaluating effects of omega-3 supplementation in people with autistic spectrum disorder, indicated that only some of them gave evidence that the supplementation improved core symptoms of autism. Much better effects (both in social and behavioral outcomes) were obtained when omega-3 acids were administered concomitantly with vitamin D [178]. In view of the above divergent findings, it is possible that the response to the intake of PUFAs may be different between subgroups of patients with autistic spectrum disorder and may depend on the etiology of the disease (i.e., genetic versus non-genetic) as well as whether the person is high- or low-functioning. All authors of meta-analyses have underlined a great divergence in the methodology used, including individual randomized clinical trials, low sample size, differences in FA doses and their combinations (omega-3, omega-6, omega-9 FAs), their co-administration with other dietary supplements (e.g., vitamin D), treatment duration, inherent individual variations among study participants, severity of the disease, existence of comorbidities, use of pharmacotherapy (for autism and/or other diseases), assessment tools, and outcome measures. The results of the most important studies, which were carried out during the last five years, evaluating the effects of omega-3 FA intake in children and adolescents are presented in Table 3.

Although for the time being, there is no clinical recommendation to supplement autistic patients with PUFAs as a dietary intervention for their disease, according to the preliminary clinical guidelines for children with autistic spectrum disorder developed by Chang and Su [154], the recommended dosage of DHA and EPA should be 1300–1500 mg/day, with a recommended duration of the supplementation 16–24 weeks. Some authors claim that a longer period of omega-3 FA supplementation is necessary because, according to literature data, it takes at least four months to observe any effect of PUFAs intake on cognitive performance [154].

Donovan and colleagues [179] wanted to check whether the intake of omega-3 acids during pregnancy and lactation has any effect on neurodevelopment in children, including the risk of autism. Based on the outcomes of 33 publications included in the review, the authors were unable to find a significant relationship between omega-3 FA supplementation in mothers and the development of autism in their children [179]. The results of these studies were inconclusive, partially due to the insufficient number of participants. In particular, participants with lower socioeconomic status as well as racially and ethnically diverse populations were not sufficiently represented. The same group of authors reached similar conclusions in another study [93]. Interestingly, Bragg et al. [180] suggested that a prenatal diet rich in FAs may act as a protective modifier of the association between environmental exposure to air pollutants, endocrine-disrupting chemicals, pesticides, or heavy metals and an increased risk of autism spectrum disorders.

### 8.3. Depression

Depression in children and adolescents may manifest as the following symptoms: feeling sad and hopeless, having no joy when doing fun things, presenting changes in sleeping or eating patterns, being tired and restless, being unable to pay attention, self-destructive or self-injury behavior, and feeling useless, worthless, and guilty. Several studies have shown that the levels of EPA, DHA, and total omega-3 FAs are significantly lower in patients with depression, and their supplementation may improve symptoms [181,182,183]. Both omega-3 and omega-6 PUFAs are involved in hippocampal neurogenesis. They influence signaling dependent on brain-derived neurotrophic factor, cyclic adenosine monophosphate response element-binding protein, and CaM, that is, pathways that play a significant role in the development of depressive disorders. PUFA deficiency is associated with changes in glutamate, serotonergic, norepinephrine, and dopaminergic neurotransmission as well as in the hypothalamic–pituitary–adrenal axis. Furthermore, omega-6 FAs are pro-inflammatory, and a bidirectional relationship between mood disorders and inflammation has been described [181]. However, most of these studies were conducted on adult patients. As the brains of children and adolescents are still developing and are more vulnerable to environmental factors and stressors, children have different nutritional needs; therefore, it is not possible to directly extrapolate the results obtained in the adult population. A recent matched case–control study of adolescents diagnosed with major depressive disorder in children conducted by Osuna et al. [184] demonstrated that a higher level of omega-3 FAs in red blood cells is correlated with a lower risk of developing major depressive disorder in children, whereas a higher omega-6/omega-3 FA ratio is correlated with a higher risk of developing major depressive disorder. According to Haberling et al. [185] and Chang and Su [154], even though the number of studies in children and adolescents is not high, outcomes of several randomized, controlled trials carried out in minors indicated beneficial effects of omega-3 FAs in pediatric depression. The increased intake of omega-3 FAs resulted in a small-to-medium effect in relation to symptom remission or reduction in depression severity when compared with placebo-treated groups [154,185]. The results of the most important studies that were carried out during the last five years, evaluating the effects of omega-3 FA intake on pediatric depression, are presented in Table 3. In the most recent review, considering seven studies with 541 patients, Thakur et al. [186] found it difficult to determine the clinical use of omega-3 FAs for depression in children and adolescents owing to the high heterogeneity in outcomes. In four of the seven studies, the improvement in depression symptoms was statistically significant. Differences in inclusion and diagnostic criteria, presence of comorbidities, treatment strategies (with or without standard pharmacotherapy), and assessment scales may be responsible for these divergent results [186]. According to the Preliminary Clinical Guidelines for Children with Major Depressive Disorder developed by Chang and Su [154], the recommended dosage of DHA and EPA is 1000 mg/day for children between 6 and 12 years of age and at least 2000 mg/day for children over 12 years old. The DHA to EPA ratio was 1:2. The recommended duration of supplementation is 12–16 weeks [154].

Further high-quality trials are required to evaluate the potential role of PUFAs in the management of neurodevelopmental conditions. These clinical studies should be multicenter and should include more participants and patient stratification. A comparable methodology should be applied, particularly related to both the quality and quantity of omega-3 and omega-6 acids, the duration of the intervention, and concomitant administration of standard pharmacotherapy.

This review concisely describes the relevance and efficacy of PUFAs in the management of three neurodevelopmental conditions in children: ADHD, autism, and depression. Our opinion is similar to that of other scientists testing and/or evaluating the potential of PUFAs in the above-mentioned conditions as a novel treatment option; that is, their effects are either mild or statistically insignificant. Although amelioration of some behavioral symptoms in pediatric patients participating in clinical trials has often been reported by both parents and clinicians, meta-analyses and systematic reviews do not present an unequivocal conclusion. Consequently, there is still no clarity or confidence regarding the use of PUFAs for neuropsychiatric disorders. However, most probably, the problem is not in the “ambiguous” effect of PUFAs but in the quality and diversity of trials/studies that brought highly variable outcomes. Therefore, better-quality research with a unified methodology is needed depending on the tested conditions: inclusion/exclusion criteria, applied doses (both cumulative PUFAs doses and doses of individual acids), treatment period, sample size, severity of a given condition, comorbidities, concurrent treatment, dietary status (potential dietary deficiencies), and measurement tools. First, it is necessary to establish an optimal dose of EPA and DHA as well as their ratio for each neurodevelopmental condition. Moreover, it is necessary to investigate whether supplementation with PUFAs would be sufficient as a single therapeutic strategy or whether it should be treated as an additional regimen to standard pharmacotherapy. Based on the outcomes of clinical trials, we can conclude that PUFAs are safe and well tolerated by the general population, including more vulnerable patients such as children, elderly people, and pregnant women.

## 9. Broader Conclusions

This review meticulously explores the multifaceted impact of fatty acid-rich diets on central nervous system development, underscoring the indispensable role of fatty acids, particularly omega-3 polyunsaturated fatty acids, in neurodevelopmental processes. The evidence presented illustrates not only the foundational significance of these nutrients in the formation and maintenance of neuronal membranes but also their profound influence on neurogenesis, synaptic plasticity, and neuroinflammation modulation.

Our discussion highlights critical research gaps, particularly in understanding nuanced interactions between dietary fatty acids and neurodevelopmental outcomes. While the current literature provides a robust framework for appreciating the beneficial effects of fatty acids, it also calls attention to the need for more nuanced investigations, especially concerning the dosage, timing, and long-term effects of fatty acid supplementation.

The broader implications of our findings suggest a pivotal role for dietary guidelines in optimizing neurodevelopmental health. It is clear that ensuring adequate intake of essential fatty acids, particularly during critical periods of development, is not only beneficial but also necessary for fostering optimal neurological and cognitive outcomes. This realization not only has implications for dietary recommendations but also underscores the importance of accessible nutritional education and interventions, especially in populations at risk of malnutrition or those experiencing dietary deficiencies.

This field stands on the cusp of significant advancements. Longitudinal studies are needed to determine the long-term effects of fatty acid intake on neurodevelopmental trajectories. Such research is crucial for developing targeted nutritional interventions that could mitigate the risks of neurodevelopmental disorders and enhance cognitive and neurological health across the lifespan.

In conclusion, this review not only consolidates current knowledge on the impact of fatty acids on CNS development but also sets a compelling agenda for future research. By bridging the gaps identified and responding to the complex questions raised, the scientific community can move closer to unraveling the intricate nexus of nutrition, neurodevelopment, and health, paving the way for innovations in dietary strategies and public health policies aimed at nurturing the full potential of future generations.

## Figures and Tables

**Table 1:** Selected dietary sources of ALA, DHA, and EPA [19,25].

Food	PUFAs Content (g/100 g of Product)		
	ALA	DHA	EPA
Seeds and nuts			
Walnuts	9.05	-	-
Chia seeds	17.81	-	-
Flaxseed	19.4	-	-
Oils			
Canola	7.45	-	-
Soybean	6.62	-	-
Olive	0.65	-	-
Fish			
Salmon, Atlantic	-	1.45	0.69
Herring, Atlantic	-	1.09	0.9
Sardines	-	0.86	0.5
Seafood			
Lobster	0.05	0.08	0.12
Shrimps	-	0.14	0.14
Scallops	-	0.10	0.07

ALA, α-linolenic acid; DHA, docosahexaenoic acid; EPA, eicosapentaenoic acid; PUFAs, polyunsaturated fatty acids.

**Table 2:** Influence of nutritional supplements on cognitive development in children and variability observed in randomized controlled trials.

Type of Study	Participants	Intervention	Main Outcomes	Ref.
Single-center, double-blind, placebo-controlled randomized clinical trial	Mother–child cohort, 736 women at pregnancy week 24, 654 participants	The pregnant women received four 1 g capsules of fish oil per day, providing 2.4 g/day of omega-3 LC-PUFA (55% EPA and 37% DHA) or four capsules with olive oil (72% *n*-9 oleic acid and 12% omega-6 LA; control group)	Maternal administration of omega-3 LC-PUFA (EPA and DHA) during pregnancy was correlated with improved early language development and reduced emotional and behavioral issues in children at 6 years of age, with particular cognitive development advantages observed in male children at 2.5 years.	[102]
Randomized, controlled trial	Mother–child pairs, 622 participants	Pregnant women in their 18–22 week of gestation received 400 mg/day of algal DHA or a placebo mixture of corn and soybean oil through delivery	Maternal fatty acid desaturase 2 (FADS2) single nucleotide polymorphisms rs174602 may modify the effect of prenatal DHA supplementation on child cognitive development at 5 years.	[103]
Multicenter, randomized, double-blind, placebo-controlled trial	Pregnant women and their children, 311 participants	Pregnant women received either a modified fish-oil (FO) preparation (500 mg DHA + 150 mg EPA/day), 5-methyl-tetrahydrofolate (5-MTHF) (400 g/day), a combination of both supplements (FO + 5-MTHF), or placebo, from gestational week 20 until delivery	No definitive impact of prenatal omega-3 supplementation on processing speed was observed in children up to 9 years old.	[104]
Subgroup analyses of a randomized trial	Infants born <29 weeks of gestation, 227 participants	Breastfeeding mothers consumed either six capsules, each containing 500 mg of DHA-rich tuna oil (to achieve a breastmilk DHA concentration of approx. 1% of total fatty acids) + a dietary supplement that provided approx. 60 mg/kg/day of DHA (high-DHA group) or 500 mg soy oil (which does not alter the fatty acid composition of the breastmilk) + a dietary supplement that provided approx. 20 mg/kg/day of DHA (standard-DHA group)	High-dose DHA supplementation in preterm infants did not demonstrate a clear benefit to IQ.	[105]
Double-blind, randomized, controlled trial	Healthy term infants, 420 participants	Infants received either fish oil (containing at least 250 mg of DHA and at least 60 mg of EPA) or placebo (olive oil) daily from birth to 6 months of age	Supplementation with fish oil from birth to 6 months did not confer significant cognitive benefits at 6 years of age.	[106]
Triple-blind, randomized, controlled clinical trial	Children with uncomplicated severe acute malnutrition (SAM), 2758 participants	Children with severe acute malnutrition were treated with three RUTF variants, i.e., DHA-HO-RUTF, HO-RUTF, and S-RUTF, in a clinical trial.	Children with severe acute malnutrition showed cognitive improvement after treatment with DHA-enriched therapeutic food.	[107]
Randomized, controlled trial	Children aged 15 months to 7 years, 1059 participants	Children received supervised isocaloric servings (≈1300 kJ, five mornings each week, 23 weeks) of a new food supplement (NEWSUP, high in plant polyphenols and omega-3 FAs, within a wide variety and high fortification of micronutrients and a high protein content), a fortified blended food (FBF) used in nutrition programs, or a control meal (traditional rice breakfast)	Nutrient-rich supplementary feeding improved cognitive function in undernourished children.	[108]
Randomized, controlled trial	Healthy children aged 4–6 years, 205 participants	Children received three prepared meals weekly for 16 weeks, containing either approximately 50 g of Atlantic salmon or 50 g of meat per meal	Moderate consumption of fish was related to better performance in specific fluid intelligence tests but did not affect overall IQ in preschoolers.	[109]
Multicenter, blinded, parallel-group, randomized, controlled trial	Children born before 29 week’s gestation, 656 participants	Children received an enteral intervention emulsion that provided 60 mg of DHA per kilogram of body weight per day or a control emulsion that provided contained no DHA from the first 3 days of enteral feeds until 36 weeks of postmenstrual age or discharge home, whichever occurred first	The use of enteral emulsion containing DHA until 36 weeks of postmenstrual age was associated with moderately higher full-scale IQ (FSIQ) scores at the age of 5 years compared to control feeding.	[53]
Prospective, randomized, double-blind study	Children aged 6 years, 108 participants	Infants received up to 18 months of life a standard infant formula (SF) or experimental infant formula (EF) enriched with milk fat globule membrane (MFGM), LC-PUFAs, and synbiotics, and a reference group of breastfed (BF) infants were also recruited	An infant formula enriched with nutrients such as MFGM, LC-PUFAs, and synbiotics led to better cognitive outcomes compared to standard formula or breast milk.	[110]
Cross-sectional study	Children aged 6–8 years, 487 participants	Parents recorded all food and drinks consumed by their children at home, at school, in afternoon care, and elsewhere outside home using household or other measures	No consistent relationship was found between dietary FAs and cognitive performance in children aged 6–8 years.	[111]
Randomized, controlled trial	Children 8–9 years old, 198 participants	Children consumed 375 g/week of oily fish or poultry (control) for 12 ± 2 week	Weekly consumption of oily fish improved cognitive functions, particularly attention and cognitive flexibility in children aged 8–9 years.	[112]
Cross-sectional study	Children aged 8–9 years, 199 participants	None	Performance in particular cognitive domains did not consistently correlate with omega-3 LC-PUFAs levels, with the exception of processing speed metrics, where all indications suggested quicker cognitive processing associated with higher omega-3 PUFAs status.	[113]
Follow-up of double-blind, randomized clinical trial	Children aged 9.5–10 years, 85 participants	Mother’s supplementation with fish oil	Pregnant mothers who supplement with fish oil may influence resting-state network function in school-aged children and generate long-lasting impacts on their cognitive processing.	[114]
Double-blind, placebo-controlled, randomized trial	Children aged 7–12 years, 106 participants	Children received either 300 mg/d of DHA or placebo for 6 months	DHA supplementation did not improve executive functions in school-aged children.	[115]
Randomized, controlled trial	Children aged 8–14 years, 119 participants	Children consumed either 0.6 L/day of a fortified milk beverage containing vitamins (A, B complex, C, D, and E), minerals (calcium, phosphorus, and zinc), fish oils (with high levels of DHA and EPA), oleic acid, and carbohydrates (sugar and honey) or 0.6 L/day of regular full milk every day for 5 months.	Fortified milk beverages with micronutrients and PUFAs appeared to support the cognitive development in children aged 8–14 years.	[116]

5-MTHF, 5-methyl-tetrahydrofolate; BF, breastfed; DHA, docosahexaenoic acid; EF, experimental infant formula; EPA, eicosapentaenoic acid; FADS2, fatty acid desaturase 2; FBF, fortified blended food; FO, fish oil; HO, high-oleic; LC-PUFAs, long-chain polyunsaturated fatty acids; MFGM, milk fat globule membrane; NEWSUP, new food supplement; PUFAs, polyunsaturated fatty acids; RUTF, ready-to-use therapeutic food; SAM, severe acute malnutrition; SF, standard infant formula.

**Table 3:** Omega-3 fatty acid intake and its effects on ADHD, autism, and mood disorders in children and adolescents: recent studies.

Type of Condition	Type of Study	Participants	Intervention	Main Outcomes	Reference
ADHD	Open-label	Children 7–15 years old, 40 participants	All participants received a combination of methylphenidate (1 mg/kg/day) and EPA (70 mg/day) + DHA (250 mg/day) for 1 month	Significantly increased quality of attention, improvement of ADHD core symptoms, significantly increased levels of EPA and DHA, significantly decreased levels of several omega-6 FAs (including arachidonic acid), and slightly decreased omega-6/omega-3 index slightly decreased. No severe side effects.	[136]
None	Randomized, open-label	Children 9–10 years old, 132 participants (57 males, 75 females)	Each group with 66 subjects. DHA-enriched fish oil capsules containing 403 mg of DHA or a daily midday lunch snack comprising 100 g of a lightly grilled fish (grouper, seabream, kingfish, emperor, or snapper) sandwich with some vegetables (providing 150–200 mg of DHA) 5 days in a week for 12 weeks	Fish oil supplement increased the DHA level more profoundly than the meal. Improvement in verbal fluency and executive functioning was noted in all children, but a significantly greater effect was seen only with executive functioning in the group receiving the DHA supplement.	[138]
Mild to moderate ADHD	Randomized, double-blind, controlled for 6 months + open-label for 6 months	Children 6–12 years old, 160 participants (118 males, 42 females)	Intervention group 79 subjects): 2 capsules/day (each capsule containing 279 mg of EPA, 87 mg of DHA, and 30 mg of GLA) for 6 months; Control group (81 subjects): 2 capsules of placebo for 6 months; Open-label phase: All children were given: 2 capsules/day (each capsule containing 279 mg of EPA, 87 mg of DHA, and 30 mg of GLA) for 6 months	Omega-3/6 dietary supplementation was not significantly correlated with the clinical improvement in ADHD symptoms or with essential fatty acids blood levels.	[139]
ADHD	Randomized, double-blind, controlled	Children 7–14 years old, 50 participants (46 males, 4 females)	Intervention group (25 subjects): 2 soft gelatin pearls/day providing a dose of 500 mg of algal DHA for 6 months; Control group (25 subjects): 2 pearls/day containing 500 mg of wheat germ oil with vitamin E (placebo) for 6 months	DHA supplementation had no beneficial effect on the symptoms of ADHD, but it had small positive effects on other behavioral and cognitive difficulties related to ADHD, such as psychosocial functioning, emotional problems, and focused attention.	[140]
ADHD	Randomized, double-blind, controlled	Children 6–18 years old, 92 participants (79 males, 13 females)	Intervention group (48 subjects): 1.2 g/day of EPA for 12 weeks; Control group (44 subjects): placebo (1.2 g/day of soybean oil) for 12 weeks	EPA supplementation improved focused attention and vigilance.	[141]
ADHD	observational cohort study	Children 6–16 years old, 60 participants (42 males, 18 females)	Intervention groups: 1st group received Mediterranean diet for 8 weeks (19 subjects); 2nd group received 4 soft gels/day providing 550 mg of EPA and 225 mg of DHA/day for 8 weeks (29 subjects); 3rd group received Mediterranean diet and 4 soft gels/day providing 550 mg of EPA and 225 mg of DHA/day for 8 weeks (19 subjects); Control group (19 subjects): Usual diet for 8 weeks	Supplementation of EPA and DHA is associated with less marked impulsive behavior in children with ADHD. A Mediterranean diet may improve the Barratt Impulsiveness Scale score, although obtained results were not conclusive in the studied population.	[142]
ADHD	Randomized, double-blind, controlled	Children 6–15 years old, 162 participants (127 males, 35 females)	Intervention group (77 subjects): for 3 months capsules containing: (1) For children 6–8 years old 336 mg of EPA + 84 mg of DHA/day; (2) For children 9–11 years old 504 mg of EPA + 126 mg of DHA/day; (3) for children 12–15 years old 672 mg of EPA + 168 mg of DHA/day. Control group (44 subjects): placebo for 3 months	No beneficial effect of omega-3 supplementation was detected.	[143]
ADHD	Randomized, double-blind, controlled	Children 6–12 years old, 60 participants (49 males, 11 females)	All participants were taking methylphenidate at a dose of 10 mg/day (in 2 doses) and 20–30 mg/kg/day (in 2 doses) from the second week. Intervention group: 1 capsule and from the 2nd week 2 capsules containing 180 mg of EPA + 120 mg of DHA/day for 8 weeks; Control group: 1 capsule and from the 2nd week 2 capsules of placebo for 8 weeks	No beneficial effect of omega-3 supplementation was detected.	[144]
None	Randomized, double-blind, controlled	Children 10 to 16 months at enrollment, born at 35 weeks’ gestation, 377 participants (195 males, 182 females)	Intervention group (189 subjects): dissolvable, 200 mg microencapsulated DHA and 200 mg of arachidonic acid powder/day for 180 days; Control group (188 subjects): placebo (400 mg of a daily microencapsulated corn oil powder) for 180 days	No overall treatment effect of DHA and arachidonic acid supplementation on caregiver-reported outcomes of child competence and problem behaviors were observed. Children in the intervention group had a decreased risk of clinical concern for autistic spectrum disorder compared with the placebo-treated group.	[145]
None	Randomized, double-blind, controlled	Children 18–38 months of calendar age who were born at ≤29 completed weeks’ gestation, 31 participants (21 males, 10 females)	Intervention group (15 subjects): oral omega 3-6-9 FA supplementation in the form of a lemon-flavored fish and borage oil (706 mg total omega-3 FAs 338 mg EPA, 225 mg DHA; 280 mg total omega-6 FAs: 83 mg GLA; and 306 mg total omega-9 FAs (oleic acid)/day for 90 days. Control group (16 subjects): Placebo (canola oil—124 mg palmitic acid, 39 mg stearic acid, 513 mg LA, 225 mg ALA, 1346 mg oleic acid/day) for 90 days	Omega 3-6-9 supplementation had beneficial effects on anxious and depressed behaviors, internalizing behaviors, and interpersonal relationship adaptive behaviors. No effects were observed on other aspects of behavior or sleep.	[146]
Autism	Randomized, double-blind, controlled	Children 5–15 years old, 54 participants (39 males, 15 females)	Intervention group (28 subjects): 1 capsule with omega-3 FAs (180 mg of EPA + 120 mg of DHA) for 8 weeks; Control group (26 subjects): 1 capsule of placebo (medium chain triglyceride) for 8 weeks	Omega-3 supplementation improved GARS score, stereotyped behaviors, and social communication.	[147]
Autism	Randomized, double-blind, controlled	Children (2–6 years old), 70 participants (57 males, 13 females)	Children were assigned to 1 of 3 different doses of treatment (25 “low”, 50 “medium”, or 100 “high” mg/kg/day of GLA + EPA + DHA) or 1 of 3 doses of placebo. Intervention groups (37 subjects): oral Complete Omega™ supplementation in the form of lemon oil flavored fish and borage oils (providing 185 mg of total omega-3 fatty acids including 112 mg of EPA, 67 mg of DHA, 122 mg of total omega-6 fatty acids including 32 mg of GLA, and 83 mg of total omega-9 fatty acids per milliliter of supplement) for 90 days; Control groups (33 subjects): Placebo (canola oil—providing 188 mg of LA, 81 mg of ALA, and 590 mg of oleic acid per milliliter) for 90 days	Intervention increased levels of omega-3 and omega-6 FAs and reduced IL-2 levels. Omega 3-6 treatment was tolerable.	[148]
Autism	Randomized, double-blind, controlled	Children (2.5–8 years old), 117 participants (100 males, 17 females)	Intervention groups: 1st group received 4 capsules/day with vitamin D3 (2000 IU/day) for 12 months (31 subjects); 2nd group received 4 capsules/day with DHA (722 mg/day) for 12 months (29 subjects); 3rd group received 4 capsules/day with vitamin D_3_ (2000 IU/day) and of DHA (722 mg/day) for 12 months (28 subjects). Control group (29 subjects): 4 capsules/day with placebo for 12 months	Possible efficacy of the intake of omega-3 FAs alone or in combination with vitamin D in the management of core symptoms of autism spectrum disorders.	[149]
Depressive disorder (n = 31) or mixed anxiety and depressive disorder (n = 29)	Randomized, double-blind, controlled	Children (7–18 years old), 60 participants	All participants received standard antidepressant therapy. Intervention group: omega-3 fish oil emulsion providing 2400 mg of total omega-3 FAs (1000 mg EPA and 750 mg DHA, EPA:DHA ratio = 1.33:1) for 12 weeks; Control group: omega-6 sunflower oil emulsion containing 2467 mg of omega-6 LA in triacylglycerol form for 12 weeks	Significant reduction in Children’s Depression Inventory scores in the group receiving omega-3 fish oil emulsion when compared to the group receiving omega-6 fish oil emulsion. At the baseline, significantly lower concentrations of EPA and DHA levels as well as a higher omega-6/omega-3 ratio were detected.	[150]
None	Randomized, double-blind, controlled	Adolescents (second-year high school students), 267 participants	Cohort I Intervention group: 4 krill oil capsules/day (260 mg of EPA/day and 140 mg DHA/day) for 3 months; Control group: 4 placebo capsules/day for 3 months; After 3 months: Intervention group:8 krill oil capsules/day (520 mg of EPA/day and 280 mg of DHA/day) for 9 months; Control group: 8 placebo capsules/day for 9 months. Cohort II Intervention group:8 krill oil capsules/day (520 mg of EPA/day and 280 mg of DHA/day) for 12 months; Control group: 8 placebo capsules/day for 12 months	No effect of one year of krill oil supplementation on depression score and on self-esteem score was demonstrated. However, high drop-out rate and lack of compliance could have influenced the obtained results.	[151]
Depressive disorder or mixed anxiety and depressive disorder	Randomized, double-blind, controlled	Children (7–18 years old), 58 participants	All patients received standard antidepressant therapy. Intervention group: 20 mL of omega-3 FAs rich fish oil emulsion (providing 2400 mg of total omega-3 FAs: 1000 mg of EPA and 750 mg of DHA, EPA:DHA ratio = 1.33:1) for 12 weeks; Control group: omega-6 FAs rich sunflower oil emulsion for 12 weeks	Improvement of symptoms measured by the Children’s Depression Inventory in the group supplemented with omega-3 fatty acids was observed.	[152]
Major depressive disorder or depressive disorder not otherwise specified	Randomized, double-blind, controlled	Adolescents (9–21 years old)	Intervention group: 3 capsules/day with fish oil for 12 weeks (1 capsule contains 450 mg of EPA, 40 mg DHA, and 260 mg of DHA; the total daily dose of EPA + DHA was 2130 mg; EPA:DHA ratio was 1.7:1); Control group: 3 capsules of placebo/day for 12 weeks	Monotherapy with fish oil was not superior to placebo for reducing depressive symptoms in high-risk youth as assessed by the Childhood Depression Rating Scale-Revised.	[153]

ALA, α-linolenic acid; DHA, docosahexaenoic acid; EPA, eicosapentaenoic acid; FA, fatty acid; GARS, Gait Abnormality Rating Scale; GLA, gamma linolic acid; IL-2, interleukin 2.

## Abbreviations

ALA	α-linolenic acid
DHA	all-cis-docosa-4,7,10,13,16,19-hexaenoic acid
EFAs	Essential fatty acids
EPA	all-cis-5,8,11,14,17-eicosapentaenoic acid
FA/FAs	Fatty acid/fatty acids
GLA	Gamma linolic acid
LA	Linolic acid
LC-PUFAs	Long-chain polyunsaturated fatty acids
PUFA	Polyunsaturated fatty acid
TAGs	Triacylglycerols
VLCFAs	Very-long-chain fatty acids
